# RNA modulates aggregation of the recombinant mammalian prion protein by direct interaction

**DOI:** 10.1038/s41598-019-48883-x

**Published:** 2019-08-27

**Authors:** Petar Stefanov Kovachev, Mariana P. B. Gomes, Yraima Cordeiro, Natália C. Ferreira, Leticia P. Felix Valadão, Lucas M. Ascari, Luciana P. Rangel, Jerson L. Silva, Suparna Sanyal

**Affiliations:** 1 Department of Cell and Molecular Biology, Uppsala University, Uppsala, Box-596, 75124 Sweden; 2 Instituto de Tecnologia em Imunobiológicos, Bio-Manguinhos, FIOCRUZ, Rio de Janeiro, 21040-900 Brazil; 3 Faculdade de Farmácia, Universidade Federal do Rio de Janeiro, Rio de Janeiro, 21941-902 Brazil; 4 Instituto de Bioquímica Médica Leopoldo de Meis, Instituto Nacional de Ciência Tecnologia de Biologia Estrutural e Bioimagem, Universidade Federal do Rio de Janeiro, Rio de Janeiro, 21941-902 Brazil; 5 Present Address: Laboratory of Persistent Viral Diseases, Rocky Mountain Laboratories, National Institute of Allergy and Infectious Diseases, National Institutes of Health, Hamilton, MT, United States of America

**Keywords:** Prions, Protein aggregation

## Abstract

Recent studies have proposed that nucleic acids act as potential cofactors for protein aggregation and prionogenesis. By means of sedimentation, transmission electron microscopy, circular dichroism, static and dynamic light scattering, we have studied how RNA can influence the aggregation of the murine recombinant prion protein (rPrP). We find that RNA, independent of its sequence, source and size, modulates rPrP aggregation in a bimodal fashion, affecting both the extent and the rate of rPrP aggregation in a concentration dependent manner. Analogous to RNA-induced liquid-liquid phase transitions observed for other proteins implicated in neurodegenerative diseases, high protein to RNA ratios stimulate rPrP aggregation, while low ratios suppress it. However, the latter scenario also promotes formation of soluble oligomeric aggregates capable of seeding *de novo* rPrP aggregation. Furthermore, RNA co-aggregates with rPrP and thereby gains partial protection from RNase digestion. Our results also indicate that rPrP interacts with the RNAs with its N-terminus. In summary, this study elucidates the proposed adjuvant role of RNA in prion protein aggregation and propagation, and thus advocates an auxiliary role of the nucleic acids in protein aggregation in general.

## Introduction

Prion diseases such as Transmissible Spongiform Encephalopathies (TSE) are a group of infectious disorders, which irreversibly cripple the nervous system of humans and other mammals^1,2^. The agents responsible for those fatal conditions are commonly referred to as prions or prion proteins (PrP). Mammalian prions are ubiquitous proteins, which share the chemical properties of all polypeptides, but surprisingly can maintain several, marginally stable, conformational states at once. In the case of TSEs, the infectious form of the prion is called scrapie (PrP^Sc^). This disease-associated conformation is amyloidogenic and self-sustaining over time and across cell divisions^3^. In recent years the number of proteins suspected of prion-like behavior has grown significantly. Some of those clearly serve a physiological purpose and are considered as functional prions^4,5^. Others are toxic in their prion-like state and have been linked to other non-infectious, degenerative disorders, such as Alzheimer’s, Parkinson’s disease and cancer^6,7^.

While the prevalence of proteinopathies becomes more evident, the necessity to understand the prion nature and evolutionary significance grows^8,9^. The outstanding characteristic of every prion is its ability to exist in two distinct physical states at the least^10^. In the case of TSEs, conversion of the cellular prion protein (PrP^C^) to its infectious form (PrP^Sc^) is the hallmark of disease onset. The two states of the protein remain chemically identical but differ significantly in their biophysical properties. Whereas PrP^C^ is mostly α-helical, soluble and susceptive to protease digestion and chaotropic agents, PrP^Sc^ favors a β-sheet rich conformation^11–14^, which renders it amyloidogenic, partially protease-resistant and insensitive to chaotropes^1^.

The most decisive of the criteria, commonly employed in PrP^C^/PrP^Sc^ discrimination, is the ability of the oligomeric scrapie protein to recruit native PrP^C^ and induce further aggregation. This ability is at the center stage of prion self-replication and maintenance of heritable phenotypic change^15^. It is also the inspiration behind the “protein-only” hypothesis, which states that the misfolded PrP^Sc^ alone is sufficient for PrP^C^ to PrP^Sc^ conversion^16–18^. This theory has been widely accepted in the field and even a study demonstrating propagation of an infectious prion in the absence of any cofactors has been recently published^19^. Despite that, a number of reports have emerged suggesting nucleic acids as the putative cofactors for prion formation and propagation^20–23^. A recent study has characterized the interaction between PrP and nucleic acids^24^. Furthermore, studies on RNA dependent phase separation of other neurodegenerative disease-related proteins have also been reported^25,26^. Apart from demonstrating that nucleic acids could facilitate prion conversion^27–35^, we recently reported that DNA and RNA modulate the aggregation of the tumor suppressor protein p53^35–38^. It is, therefore, becoming evident that nucleic acids, particularly RNA, have pronounced effect on protein folding, misfolding and aggregation, in some cases inhibiting and in other cases stimulating the processes^39,40^.

When PrP is overexpressed in the cytoplasm of the cells treated with proteasome inhibitors, it converts to an alternative isoform and accumulates in the perinuclear organelles (aggresomes), together with RNA^41,42^. Another type of membraneless structure – an organelle-like ribonucleoprotein particle, is formed when cytosolic PrP co-aggregates with RNA^43^. RNA is also detected in highly infectious brain isolates from TSE-positive Syrian hamsters^44^. Together with the demonstrations that RNA can induce scrapie phenotype *in vitro*^45,46^ this suggests a strong possibility for macromolecular interplay between RNA and PrP, *in vivo*. Transcriptome extracts of murine neuroblastoma (N2a) cells are shown to co-aggregate with rPrP to form large, partially protease resistant complexes, *in vitro*^30,47^. Finally, Maharana *et al*.^25^ show that high protein to RNA ratios promote phase separation and aggregation of the prion-like RNA binding proteins such as TDP43 and FUS, whereas low ratios prevent it. In accordance with these findings, the emerging concept is that the amyloid fibrils arise from more dynamic liquid droplets and glassy-solid states^48,49^, where RNA acts as phase modulator^26,35,39,50^. These observations hint at an auxiliary, previously unassumed function of RNA, in addition to its traditional role in the central dogma of molecular biology.

In the present study, we investigate the role of RNA in the aggregation of the full-length, recombinant, murine PrP (rPrP^23–231^ variant, referred hereafter as rPrP) by using static light scattering (LS), dynamic light scattering (DLS), sedimentation, transmission electron microscopy (TEM) and circular dichroism (CD). Furthermore, we follow the aggregation kinetics of rPrP, alone and in the presence of various RNAs, by monitoring the time-dependent increase in LS in a stopped-flow instrument. Our results suggest a modulatory role of RNA in rPrP aggregation, where high protein to RNA ratios lead to the formation of large amorphous aggregates of rPrP, whereas low ratios prevent it, and in turn, generate soluble, oligomeric species that can seed *de novo* rPrP aggregation. Furthermore, we observe that RNA co-aggregates with rPrP and gets partial resistance to RNase digestion. We therefore propose that RNA is an active player in rPrP aggregation, capable of modulating the outcome by direct interaction.

## Results

### Bimodal action of RNA on the aggregation of rPrP studied by LS and DLS

Equilibrium aggregation of rPrP (5 μM) alone or in the presence of various RNAs and ribonucleoside tri-phosphates (rNTPs) was studied by recording LS at 400 nm after incubating the samples for at least 10 minutes at room temperature. The RNAs include *in vitro* transcribed mRNAs for dihydrofolate reductase (DHFR) from *Escherichia coli* and Green Fluorescent Protein + (GFP+), domains V, IV and II from 23S ribosomal RNA (rRNA) of *E*. *coli* and bulk-tRNAs isolated from *E*. *coli* MRE600.

**Figure 1:**
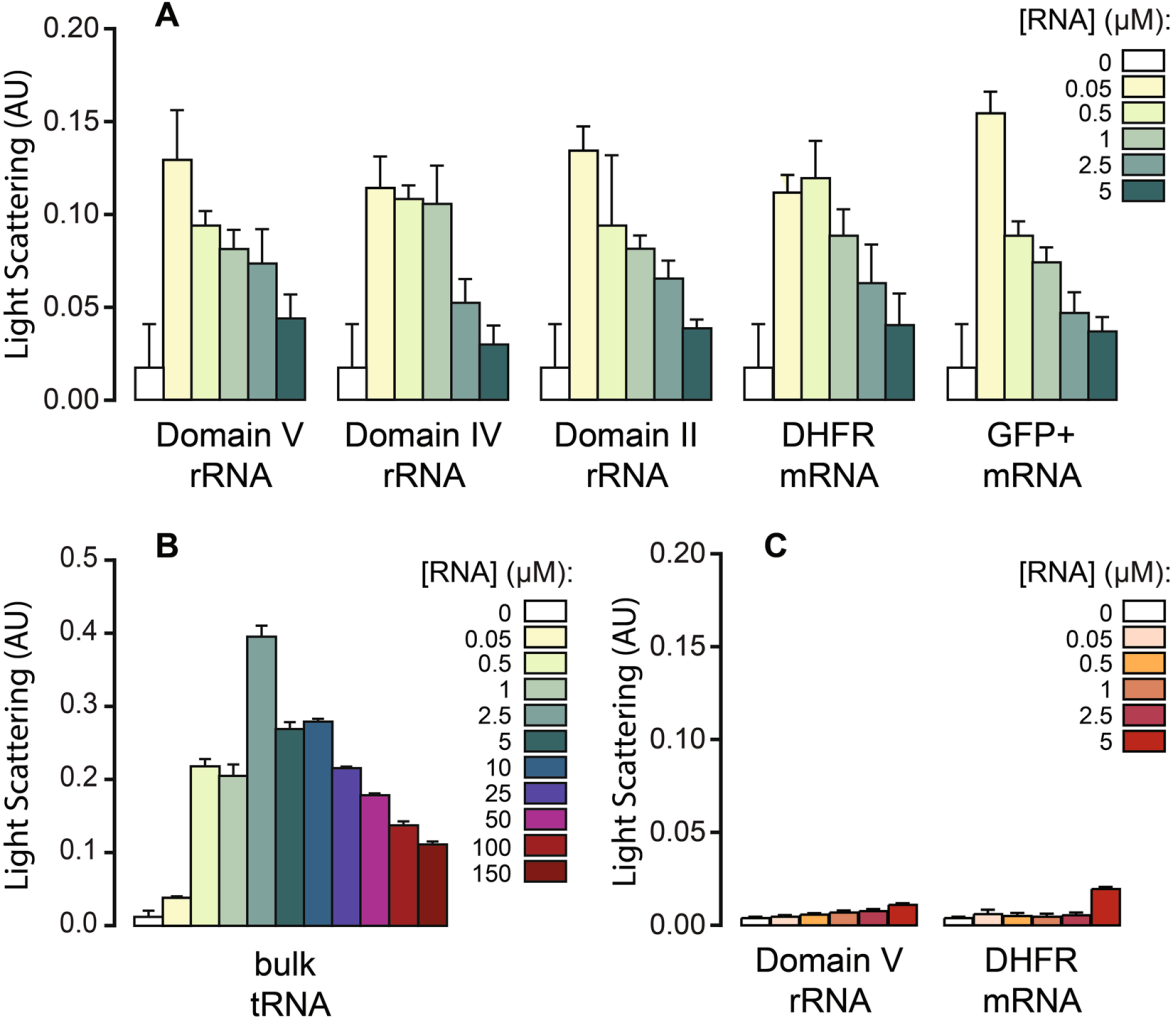
Equilibrium aggregation of rPrP (**A**,**B**) and rPrP^90–231^ (**C**) in the presence of various RNAs (as indicated) followed by LS at 400 nm in a steady state fluorimeter. Aggregation was achieved by incubating the protein (5 μM) without or with RNAs for 10 minutes at the room temperature without agitation. AU – arbitrary units.

All rRNA domains and mRNAs tested here stimulated aggregation of rPrP in a concentration dependent manner (Fig. 1A). At low RNA concentration the LS signal increased over ten-fold compared to the rPrP only sample. With increase in RNA concentration, however, the aggregation decreased gradually (Fig. 1A). This phenomenon is analogous to the recently reported effect of RNA on the aggregation of the central core domain of the tumor suppressor protein p53^36^. However, unlike p53C, at the highest RNA concentration tested here, LS values still remained ~two-fold higher than the protein only reaction. Similar results obtained with different RNAs also suggest that RNA interaction for rPrP aggregation is not dependent on any particular sequence or secondary structure.

Interestingly, tRNAs showed the strongest impact on the aggregation of rPrP among all RNAs tested here. Even though a relatively higher concentration of tRNAs was needed to stimulate rPrP aggregation, the addition of tRNAs led to a 20-fold increase in rPrP aggregation (Fig. 1B). Despite that, the overall pattern of tRNA induced rPrP aggregation remained similar to that of other RNAs. After the initial spike in aggregation with 2.5 μM tRNA, LS gradually decreased with increase in tRNA concentration. Similar to what we observed for p53C^36^, free rNTPs up to a rPrP to rNTP ratio = 1:100 exerted no effect on the aggregation of rPrP (data not shown). Thus, we conclude that the modulation of rPrP aggregation is a function of the structured RNAs.

To investigate the role of the unstructured N-terminal region of the prion protein, we subjected a truncated variant of rPrP (rPrP^90–231^), which lacks the octapeptide repeats in the N-terminal domain, to the aggregation assay in the presence of DHFR mRNA and domain V rRNA. In comparison to rPrP, rPrP^90–231^ aggregated to a much lesser extent on its own, as reflected by the LS values (Fig. 1C). More interestingly, neither of the two RNAs tested, showed any influence on the aggregation of rPrP^90–231^. This result is indicative of the importance of the N-terminus of rPrP in nucleating its aggregation and also confirms that RNA interactions involve the N-terminus of rPrP, as proposed earlier^30^.

We also evaluated the hydrodynamic radius (Rh) and size distribution of the rPrP aggregates that are formed in the presence of low concentration of RNA (rPrP:RNA = 1:0.05) by employing DLS. Regardless of the type of RNA, all aggregated species displayed low polydispersity and yielded good correlation functions, demonstrating high sample homogeneity. The Rh values of different samples were on average ~100 nm, which indicated the presence of high molecular weight aggregates (Table 1). Interestingly tRNA containing samples formed significantly larger aggregates, with Rh value ~700 nm, approximately seven times larger than those with other RNAs. This result corroborates with our LS data where highest level of aggregation of rPrP was seen with tRNA (Fig. 1B). At high RNA concentration (rPrP:RNA = 1:2), the samples were polydisperse with no indication of large aggregates.

**Table 1:** Mean, cumulant R_h_ (hydrodynamic radii) and polydispersity values estimated by DLS for rPrP (5 μM) aggregation reactions with different RNAs, at a protein to RNA ratio of 1:0.05.

Sample	R_h_ (nm)	Polydispersity (%)
rPrP + Domain IV rRNA	100.98 ± 5.64	7.4
rPrP + Domain V rRNA	81.34 ± 1.64	12.8
rPrP + DHFR mRNA	100.46 ± 4.95	12.7
rPrP + GFP + mRNA	103.69 ± 4.85	19.3
rPrP + bulk-tRNA	700.68 ± 60.9	6.1

### Fast kinetics analysis of the RNA induced and seeded aggregation of rPrP

**Figure 2:**
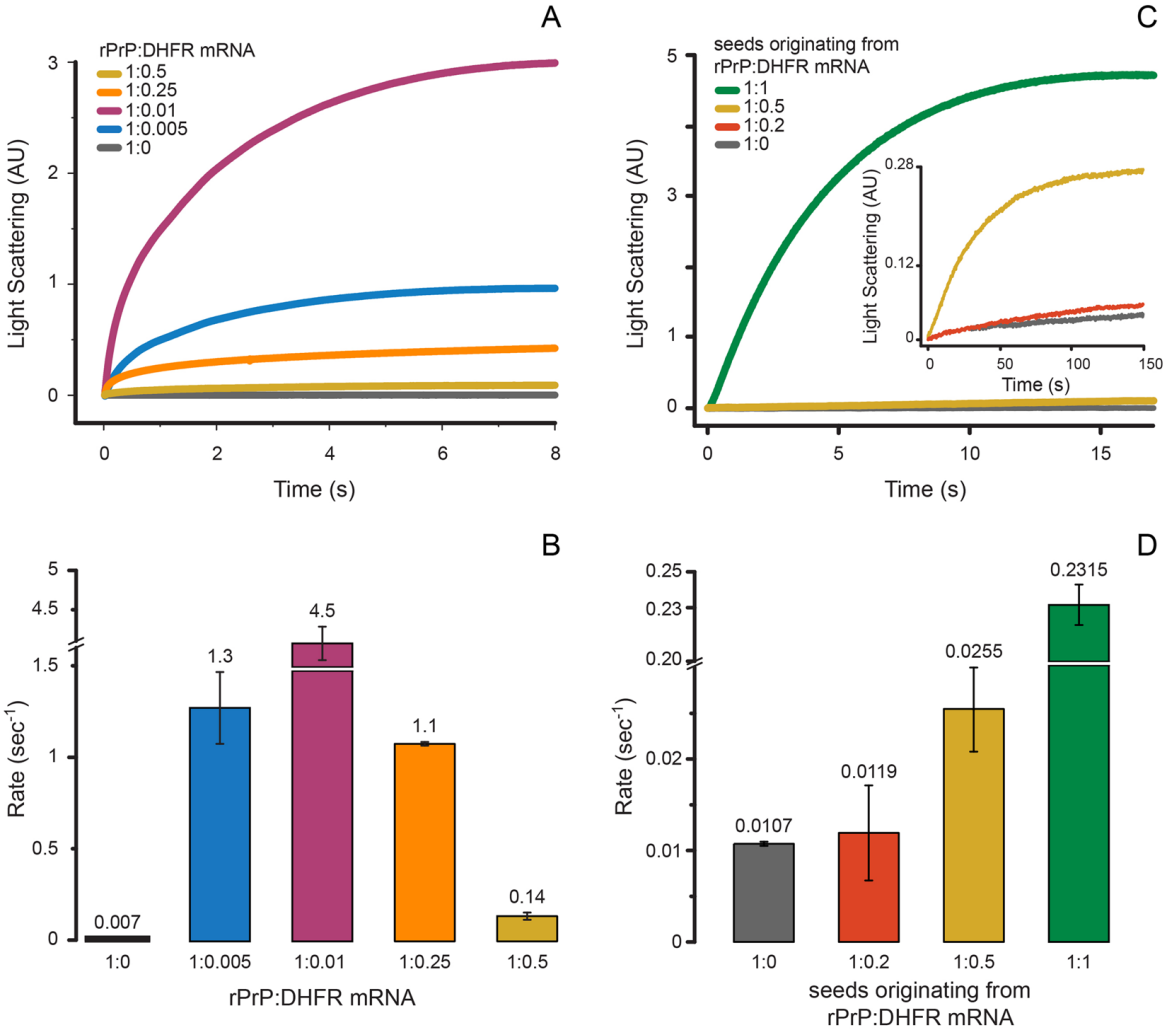
Fast kinetics of rPrP aggregation at room temperature followed by LS in a stopped-flow instrument. (**A**) Time course and (**B**) rates of aggregation of rPrP (2 μM) alone or with DHFR mRNA at various rPrP to RNA ratios. (**C**) Time course and (**D**) rates of rPrP (2 μM) aggregation after seeding the reaction with 10% (v/v) SN from rPrP aggregation reactions with various rPrP to DHFR mRNA ratios. The inset in (**C**) shows LS curves beyond 150 sec. AU – arbitrary units. The error bars in (**B**,**D**) indicate standard deviation from three independent measurements.

We have followed the kinetics of rPrP aggregation in the presence and absence of RNA at varying rPrP to RNA ratios. For that, we monitored the time-dependent change in LS at 400 nm after rapid mixing of rPrP and DHFR mRNA in a stopped-flow instrument. rPrP aggregated alone at a very slow rate with *k*_*obs*_ = 0.007 s^−1^ (Fig. 2A,B). Addition of very small amount of RNA, at rPrP to RNA ratio = 1:0.005, accelerated the rate of aggregation about 200 times, resulting in *k*_*obs*_ = 1.3 ± 0.2 s^−1^. The rate of rPrP aggregation peaked (*k*_*obs*_ = 4.5 ± 0.005 s^−1^) at a rPrP to RNA ratio of 1:0.01. In line with the steady-state LS measurements (Fig. 1), not only the extent but also the rate of rPrP aggregation decreased with further increase in RNA concentration. At 1:0.25 rPrP to RNA ratio the rate of rPrP aggregation decreased to 1.1 s^−1^ and finally dropped to 0.14 s^−1^ at 1:0.5 rPrP to RNA ratio. These results clearly suggest bimodal effect of RNA on rPrP aggregation, similar to the results obtained with p53C^36^.

To probe the nature of rPrP aggregates produced at different protein to RNA ratios, we designed a seeding experiment, where supernatant (SN) from rPrP aggregation reactions, prepared without or with DHFR mRNA, was treated with RNAse A and rapidly mixed with native rPrP in a stopped-flow instrument at a 10-fold dilution. The time-dependent change in LS was recorded to obtain the kinetics of seeded rPrP aggregation. SN from the protein only sample did not enhance the rate of *de novo* rPrP aggregation (Fig. 2C) (*k*_*obs*_ = 0.01 ± 0.001 s^−1^). Also, SN from low RNA reaction (rPrP to RNA = 1:0.2) did not show any stimulation of rPrP aggregation (*k*_*obs*_ = 0.02 ± 0.005 s^−1^). In contrast, SN from a high RNA reaction (rPrP to RNA = 1:0.5) increased the rate of rPrP aggregation more than two-fold (*k*_*obs*_ = 0.026 ± 0.005 s^−1^). However, a much more dramatic increase of the rate of *de novo* rPrP aggregation occurred when SN from a 1:1 rPrP to DHFR mRNA reaction was used for seeding (Fig. 2C). In this case, the rate of rPrP aggregation became 20-fold higher than the protein only reaction, with *k*_*obs*_ = 0.232 ± 0.005 s^−1^ (Fig. 2D). This result suggests that in abundance of RNA (low protein to RNA ratio) soluble, oligomeric rPrP seeds are formed, which could nucleate *de novo* rPrP aggregation.

### Quantitative analysis of rPrP aggregation in the presence of RNA by sedimentation assay

**Figure 3:**
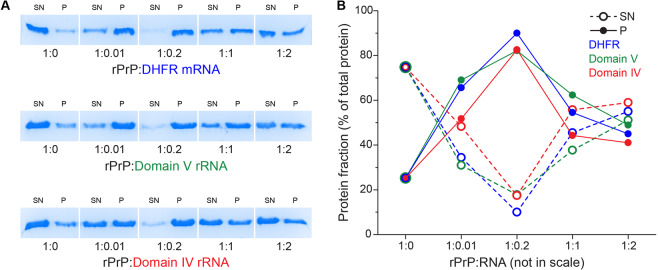
Distribution of rPrP in the supernatant (SN) and the pellet (P) fractions in the presence of RNA, assessed by SDS-PAGE. rPrP samples (10 μM) with and without RNAs at various ratios were incubated 10 min at room temperature and then centrifuged at 20,000 g. Equal volumes of supernatant and buffer equilibrated pellet were ran on a 15% SDS acrylamide gel (**A**). The amount of protein in each sample was estimated as a percentage of total protein available in each supernatant/pellet pair (**B**).

Complementary to LS, we quantified the fractions of soluble and insoluble rPrP in the presence and absence of three different RNAs by sedimentation assay with low-speed centrifugation followed by SDS-PAGE (Fig. 3A). Analysis of the relative amounts of rPrP in the SN and the pellet fractions in a rPrP only reaction (Fig. 3B) showed that only ~20% of total rPrP protein aggregates in the absence of RNA and the rest of the protein (~ 80%) remains in the soluble fraction. In stark contrast, the distribution of rPrP between the soluble and insoluble fractions reversed completely upon addition of a small amount of RNA. At the rPrP to RNA ratio at 1:0.01, ~20% rPrP remained in the soluble fraction and ~80% partitioned in the insoluble pellet. At rPrP to RNA ratio of 1:0.2, ~90% of rPrP aggregated, thereby partitioning almost exclusively to the insoluble fraction. However, with further increase in RNA, the amount of insoluble aggregated rPrP gradually decreased and the fraction of soluble rPrP increased. At the highest RNA concentration tested here (rPrP:RNA = 1:2) the distribution of rPrP between the soluble and insoluble fractions became almost equal (Fig. 3A,B). This result corroborates strongly with our LS data and strengthens the conclusion that RNA modulates rPrP aggregation in a bimodal fashion.

### Characterization of the rPrP aggregates with TEM

**Figure 4:**
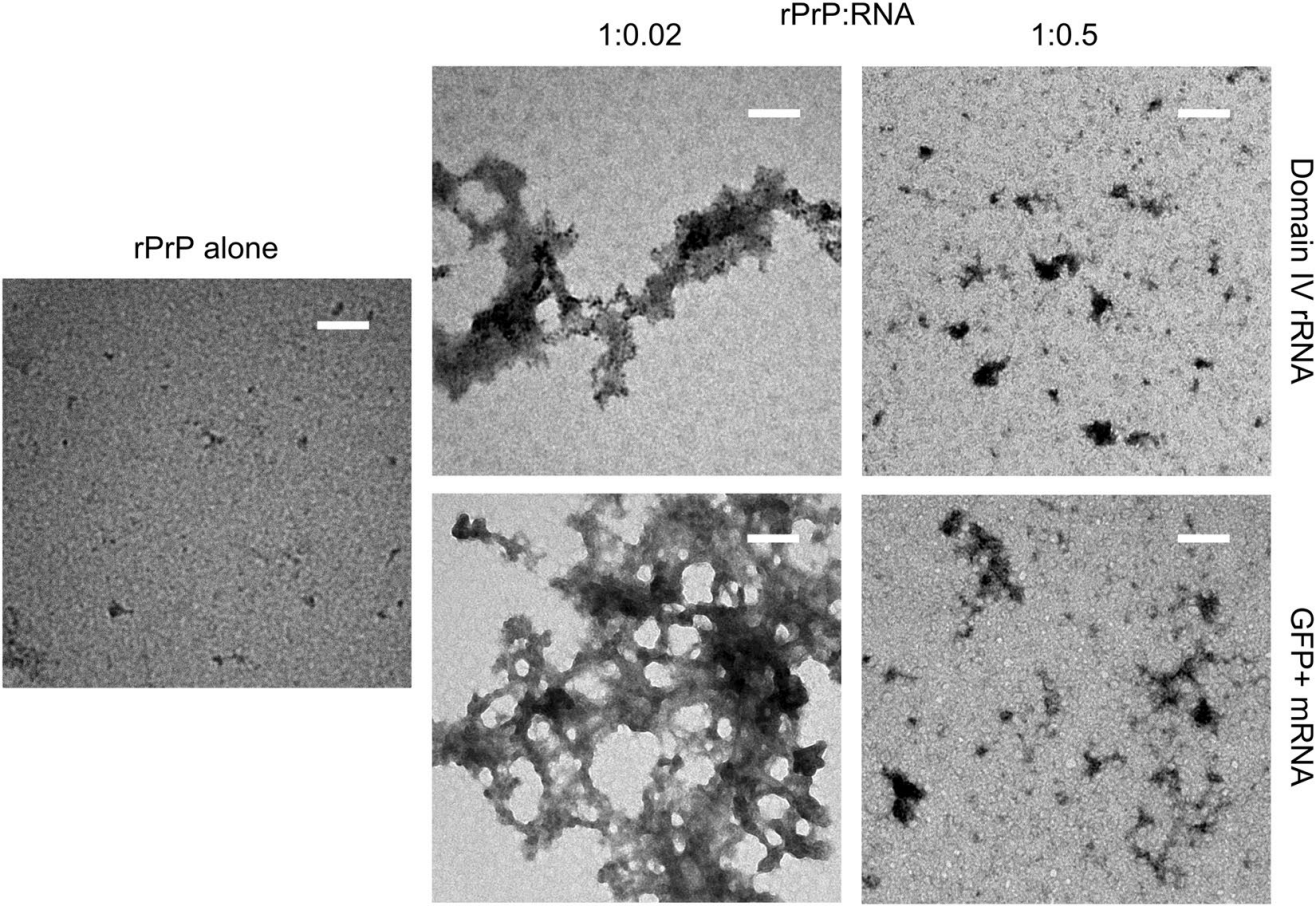
TEM images of rPrP (5 μM) obtained after 10 min incubation at room temperature in the presence of GFP + mRNA and domain IV of the 23S rRNA, at protein to RNA ratios of 1:0.02 and 1:0.5. Scale bars correspond to 100 nm.

To probe the morphology of rPrP aggregates formed in the presence and absence of RNA we subjected the rPrP samples to TEM analysis. Samples of rPrP alone or with low RNA (rPrP:RNA = 1:0.02) and high RNA (rPrP:RNA = 1:0.5) concentrations were briefly incubated at room temperature. The experiments were conducted with both GFP+ mRNA and domain IV rRNA. As shown in Fig. 4, the rPrP-only samples contained only few aggregated particles of a size range far below 100 nm. The addition of RNA in low concentration resulted in the formation of densely packed, amorphous aggregates, reaching a couple of micrometers in size (Fig. 4). In contrast, when RNA concentration was increased to obtain 1:0.5 rPrP to RNA ratio, the average size of the aggregates visibly decreased and they appeared to be distributed more evenly. In accord with previous data^31,47^, we did not detect any fibrillar structures in rPrP aggregates under our experimental conditions. Our TEM results demonstrate that in the presence of low concentration of RNA, rPrP undergoes massive aggregation - typical for liquid-liquid phase separation behavior. Alternatively, with high concentration of RNA there is only limited exclusion of rPrP from solution, which corroborates well with our LS and sedimentation data.

### Demonstration of direct RNA-rPrP interaction by co-sedimentation analysis

To test whether RNA interacts with rPrP during aggregation, we incubated rPrP with DHFR mRNA at 1:0.2, 1:0.1 and 1:0.05 rPrP to RNA ratios, which according to our sedimentation assay induce high levels of rPrP aggregation (Fig. 3A). In this experiment, we kept the RNA concentration fixed and varied rPrP concentration to achieve the desired ratios so that the sedimentation of RNA can be quantified. The SN and the pellet fractions were separated after low speed centrifugation and the RNA content was analyzed by agarose gel electrophoresis.

**Figure 5:**
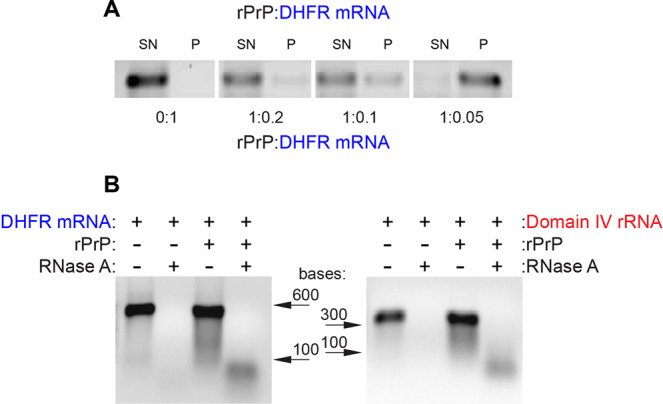
Sedimentation and RNase A digestion assays demonstrating RNA-rPrP interaction during rPrP aggregation. (**A**) Distribution of DHFR mRNA in the supernatant (SN) and pellet (P) at different rPrP to RNA ratios as assessed by agarose gel electrophoresis. For quantitative comparison, the RNA concentration was kept constant (1 μM) and rPrP concentration was varied to achieve different rPrP to RNA ratios. (see materials and methods for details). (**B**) RNase A resistance assay with DHFR mRNA (1 μM) and domain IV rRNA (1 μM) after incubation without and with rPrP (20 μM) at 1:0.05 rPrP to RNA ratio. Equal volumes of the sample were run in a 1% agarose gel.

DHFR mRNA alone (without rPrP) was perfectly soluble (Fig. 5A). However, when rPrP aggregation reactions were conducted in the low RNA conditions, RNA co-precipitated with rPrP and populated mostly in the insoluble pellet fraction. The amount of RNA in the pellet increased with decrease in RNA concentration (rPrP to RNA ratio 1:0.2 → 1:0.1 → 1:0.05). At a rPrP to RNA ratio of 1:0.05, which leads to a very high rPrP aggregation, almost all available RNA accumulated in the pellet (Fig. 5A). The experiment with domain IV of the 23S rRNA produced similar results (data not shown). These experiments suggest direct interaction between RNA and rPrP during aggregation.

To further investigate the nature of the RNA-rPrP complexes we performed RNase protection assays at rPrP to RNA ratio of 1:0.05, with both DHFR mRNA and domain IV rRNA at 1 μM concentration. The reactions were treated with 25 μg/mL RNase A for 30 minutes at 37 °C. While RNase treatment completely digested the RNAs in the absence of rPrP, partial resistance to RNase was observed for both the RNAs coaggregated with rPrP (Fig. 5B). These results indicate that the RNAs are partially protected from RNases in the rPrP aggregates.

### Characterization of RNA-rPrP interaction with CD

**Figure 6:**
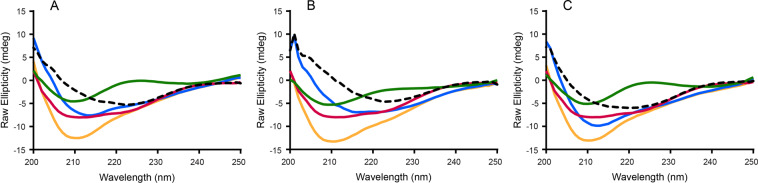
CD spectra of rPrP (5 μM) aggregated in the presence of different RNAs (**A**) domain V rRNA, (**B**) DHFR mRNA, (**C**) GFP+ mRNA, at 1:0.05 protein to RNA ratio. For each sample, three spectra were collected; protein alone (red trace); RNA alone (green); rPrP-RNA complex (blue trace). The sum of the protein alone and RNA alone spectra produced the cumulative spectra (yellow trace); RNA spectra subtracted from the complex spectra (blue trace) generated the difference spectra (dotted line).

To further elucidate the nature of interaction between RNA and rPrP during co-aggregation, we conducted CD experiments at a 1:0.05 protein to RNA ratio using domain V rRNA (Fig. 6A), DHFR mRNA (Fig. 6B) and GFP+ mRNA (Fig. 6C). The results show that in all cases the rPrP-RNA complex spectra (blue traces) and the difference spectra (complex spectra minus RNA spectra – dotted lines), diverge significantly from the spectrum of rPrP alone (red traces) (Fig. 6). They also differ from the arithmetically derived cumulative spectra for rPrP and RNA (yellow). Compared to the protein-only spectra and cumulative spectra, the rPrP-RNA complex spectra show some loss of ellipticity. Most likely the RNAs contributed to a significant decrease in the helicity of the otherwise predominantly α-helical native CD spectrum of the protein. In conclusion, it appears that RNA not only co-aggregates, but directly interacts with rPrP, causing significant conformational changes in the protein.

## Discussion

Since the description of PrP as the infectious agent behind scrapie, its biological function and implication in a variety of human proteinopathies, ranging from amyloidosis to cancer, have been reported^51^. Also, it has gradually become evident that nucleic acids are involved in the propagation of prions^40,52^. Of the nucleic acid pool available in an eukaryotic cell, RNA was categorically proven to stimulate prion protein conversion and formation of protease resistant prion fibrils^45,47,53^. RNA isolated from scrapie associated fibrils was shown to aggregate recombinant PrP to a prion-like conformation^44,46^. It was also demonstrated that certain anti-prion compounds specifically target the RNA-mediated protein folding activity of the ribosome^52,54–57^. These arguments support the proposal that one of the putative factors involved in mediating prion protein conversion is RNA.

**Figure 7:**
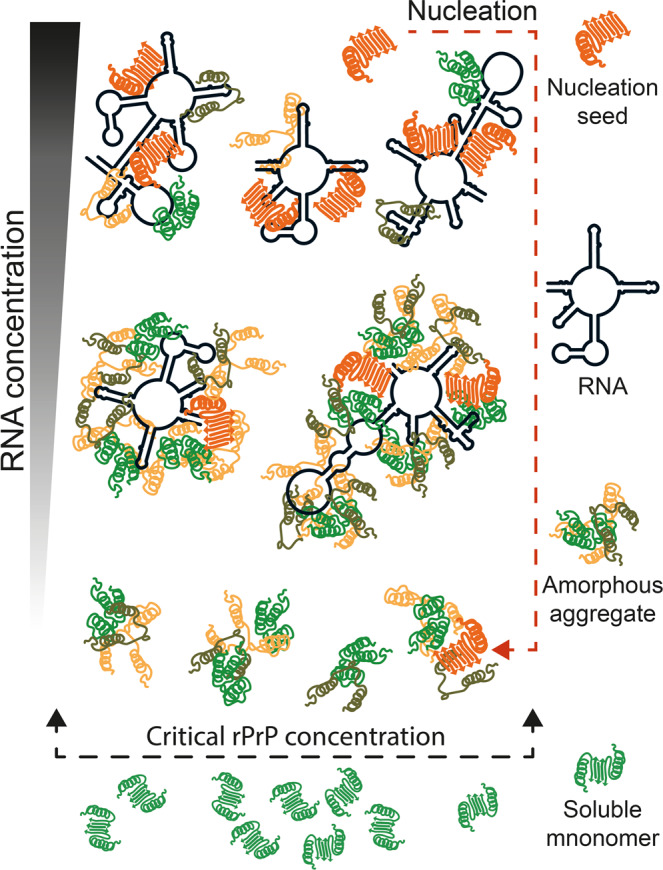
Schematic representation of the proposed effects of RNA on rPrP aggregation. Upon reaching the critical concentration rPrP forms very small aggregates in the absence of RNA. When the protein is in much excess over RNA (high rPrP to RNA ratio), rPrP aggregates profusely, sequestering the RNA. Alternatively, at low protein to RNA ratio, rPrP aggregation decreases and formation of oligomeric species, capable of nucleating *de novo* rPrP aggregation, prevails.

In the present study, we used *in vitro* transcribed RNAs to clarify the role of RNA in the aggregation of rPrP. In line with previous reports^30,31^ we observed that rPrP hardly aggregates at physiological pH and salt concentration. However, RNAs of various source and length, significantly influence rPrP aggregation in a concentration dependent manner. Small amounts of RNA, 50–100 times lower in concentration than rPrP dramatically enhance its aggregation – both in terms of the amount and the rate (Fig. 2A,B). Inversely, high concentrations of RNA, equimolar to rPrP (or higher) suppress rPrP aggregation (Figs 1A,B, 2, 3 and 4). This is a clear example of the bimodal action of RNA, observed for other prion-like systems such as p53C^36^, TDP43 and FUS^25^. In addition, SN from rPrP aggregation reactions with RNA in high concentration, promotes *de novo* rPrP aggregation, while SN from reactions with low RNA concentration does not (Fig. 2B). This result suggests that most likely oligomeric aggregates of rPrP emerge at high RNA conditions. Due to their sub-microscopic size, such species remain undetected by standard approaches such as LS, sedimentation and TEM. However, such oligomeric species are capable of nucleating *de novo* rPrP aggregation – a typical feature of prionogenic systems. This result further strengthens the proposed role of RNA in prion conversion and propagation^44–46,58^. In line with our observations we propose a schematic model of the bimodal role of RNA in rPrP aggregation, which is summarized in Fig. 7.

Another important finding of this work is that, irrespective of sequence, size or source RNA co-sediments with rPrP when added in low concentration (Fig. 5A). The RNA-rPrP association is such that a significant population of low molecular weight RNA remains protected even after extensive treatment of the aggregates with RNase (Fig. 5B). This result indicates a direct interaction between RNA and rPrP. The interaction appears to be associated with structural rearrangements in both macromolecules (Fig. 6), which supports our previously published observation that RNA alters the secondary structure of rPrP^30^. A similar result was recently reported for lysozyme aggregation in the presence of ribosomes^59^, where rRNA co-aggregated with lysozyme.

Since different RNAs tested in our study exerted analogous effect on rPrP aggregation, we speculate that the process depends more on the physical properties of RNA than on its sequence or structural motifs. As the process is virtually insensitive to free rNTPs and an inverse relationship between the size of the RNA and its effective concentration can be observed, our results highlight the significance of a defined polynucleotide surface for rPrP aggregation. On the other hand, our data suggest that rPrP interacts with RNA with a specific site. Since the N-terminally truncated rPrP variant (rPrP^90–231^) is completely insensitive to RNA (Fig. 1C), it seems likely that the unstructured N-terminal domain of rPrP is involved in RNA interaction. This is dissimilar to other proteins such as FUS and TDP-43, which interact with RNA through their prion-like domains^25^. The intrinsically disordered N-terminal domain of rPrP is referred to as the low-complexity region (LCR). Likely the LCR of rPrP has a hybrid function, acting as a mediator of RNA-based phase transition^60^. We have previously found that shortening the N-terminal domain destabilizes rPrP and promotes irreversible denaturation and aggregation^61^. The N-domain could, therefore, be a site for interaction with different ligands. While heparin binding has a stabilizing effect on it^62^, RNA binding leads to destabilization, condensation and aggregation. It remains to be established whether or not the rPrP-RNA complexes display any infectious behavior *in vivo*. There is mounting evidence showing that RNA, among other polyanions, can trigger the *in vitro* conversion of PrP into scrapie-like PrP species^58,63–65^. As long as PrP^Sc^ can be formed, it can amplify the infectious process by itself, but the role of the endogenous cofactors e.g. RNA in the propagation of infectivity of synthetic prion strains cannot be ruled out^66^. It has been described that different prion strains require different RNAs for efficient conversion^27^. However, demonstrating *de novo* infectivity using rPrP and RNA condensates is not straightforward^44,64^ and will require special laboratory set up beyond the scope of this work. One possibility would be to employ rPrP and RNA in the protein misfolding cyclic amplification (PMCA) assay under our reaction conditions, which will be a goal for future investigations.

The role of RNA in formation of membraneless structures, such as stress granules and speckles is well-established. In case of the prion-like RNA binding proteins TDP43 and FUS, it has been demonstrated that RNA induces phase separation of the proteins in a concentration dependent manner^25^. These proteins remain soluble in the nucleus – where RNA is readily available, but have higher tendency to undergo phase separation in the cytoplasm – where RNA is present in much lower concentration^25^. The significance of our data could be discussed in similar terms when asked for *in vivo* implication of the observed RNA effect on rPrP (Fig. 7). Although cellular PrP is primarily GPI-anchored to the plasma membrane, certain cell tissues appear to retain a significant fraction of endoplasmic reticulum with non-translocated PrP in the cytoplasm^67–69^. In that respect, it was already demonstrated that cytosolic PrP interacts with RNA *in vivo*, leading to the formation of large membraneless aggresomes^30,43,70^. Nonetheless, the true nature, functional or pathogenic, of cytosolic PrP-RNA condensates remains to be elucidated. In conclusion, our demonstration of the bimodal action of RNA on rPrP aggregation provides strong reinforcement and mechanistic insight to the proposed role of RNA in the processes of prion conversion^44–46,52^ and membraneless compartmentalization of the prion-like proteins^39,71^.

## Methods

### Protein expression and purification

Heterologous expression in *E*. *coli* and consecutive purification of full-length murine rPrP (rPrP^23–231^) and its N-terminally deleted variant rPrP^90–231^ was conducted by high affinity column refolding in a ÄKTA Prime Plus (GE Healthcare) as previously described^72^.

### *In vitro* transcription and extraction of RNAs

Almost all RNAs included in this study were transcribed *in vitro* with T7 RNA polymerase using PCR amplified DNA templates as described in^73^. These include mRNAs for DHFR (*E*. *coli*) and GFP + , and domains II, IV and V from 23S rRNA (*E*. *coli*). The pGEM4Z plasmid encoding the complete 23S rRNA sequence of *E*. *coli* K12^74^ was used as the template for different domains of rRNA. The templates for DHFR and GFP+ mRNAs were generated from their respective DNA clones in pET-24b (Novagen). The bulk-tRNA was extracted from *E*. *coli* MRE600 cells by phenol-chloroform treatment using laboratory protocol. All RNAs were quantified using Nanodrop™ 1000 spectrophotometer (Thermo Scientific V3.6).

### Equilibrium aggregation assays

rPrP (final concentration 5 μM unless otherwise mentioned) was diluted in 10 mM Tris-HCl (pH 7.5) and 100 mM NaCl (buffer A) prior to addition of the RNAs or rNTPs. For all equilibrium aggregation measurements such as LS, DLS, TEM and sedimentation, the reactions were incubated 10 minutes at room temperature. The rPrP stock was centrifuged at 20,000 g for 30 minutes prior to sample preparation. Aggregation assays were done without agitation or stirring.

### Light scattering – equilibrium and fast kinetics measurements

Equilibrium aggregation of rPrP and rPrP^90–231^ proteins (5 μM) was followed by Rayleigh light scattering (both excitation and emission at 400 nm) in an Infinite M200 PRO multimode microplate reader (Tecan Trading AG, Switzerland). Each experiment was repeated three times and averaged for presentation in the figures, where background LS for the various RNA concentrations was subtracted. Further, fast kinetics of rPrP (2 μM) aggregation without or with various RNAs were recorded under the same conditions in a SFM-3000 stopped-flow instrument (BioLogic Science Instruments, France) by monitoring the increase in LS for up to 150 seconds with 500 μs increments. The data were fitted with single exponential function for rate estimation.

### Seeding experiments

For seeding, rPrP (2 μM) samples at varying rPrP to DHFR mRNA ratios were incubated at room temperature for 10 minutes and then treated with 25 μg/mL RNase A (ThermoFisher Scientific) for 2 hours at 37 °C. Larger aggregates were removed by centrifugation at 20,000 g for 30 minutes. A fraction of the resulting supernatant (10% v/v) was rapidly added to a fresh preparation of rPrP (2 μM) in a stopped-flow instrument and the fast kinetics of aggregation were recorded as described above. Rates were estimated by fitting the data with a single-exponential function.

### Sedimentation assays

rPrP at a final concentration of 10 μM was incubated at room temperature without and with RNAs at various ratios for sedimentation analysis using SDS-PAGE. After 10 min, the reactions were centrifuged at 20,000 g at 4 °C and the supernatants were removed in fresh tubes. Pellets were then solubilized in buffer A and the volume was made equal to the corresponding supernatant. Further, fixed volumes of pellet and supernatant from each sample were run in 15% SDS-PAGE. After completion, gels were visualized by Coomassie blue staining and the protein bands were analyzed in pairs by Image J 1.51j8 software^75^. The bands in each supernatant-pellet pair, corresponding to each rPrP:RNA ratio, were assumed to have a combined intensity of 100%.

For quantitative estimation of RNA in pellet and supernatant, a sedimentation assay was conducted with fixed concentration of RNA (1 μM) and rPrP concentration was varied to achieve various ratios. After 10 min incubation the pellet and the supernatant were separated in the same way as described above. The pellets were re-solubilized with buffer A supplemented with 0.1% SDS and then subjected to 1% agarose gel electrophoresis. The band intensities were quantified with Image J 1.5 software^75^.

### RNase protection assay

rPrP (20 μM) was mixed with DHFR mRNA (1 μM) or domain IV rRNA (1 μM) to achieve 1:0.05 (rPrP:RNA) ratio and incubated at room temperature for 10 minutes. After that, the reactions (along with RNA only controls) were subjected to RNase digestion by incubation with 25 μg/mL RNase A (ThermoFisher Scientific) at 37 °C for 30 minutes. Then the samples were supplemented with 1 U/μL of RiboLock RNase inhibitor (ThermoFisher Scientific) and RNA was extracted by phenol-chloroform to ensure complete nuclease inactivation. The RNAs were run in 1% agarose gel and quantified with Image J 1.5 software^75^.

### Transmission electron microscopy

For TEM measurements, rPrP (5 µM) was incubated with GFP+ mRNA and domain IV rRNA to achieve rPrP:RNA ratios of 1:0.02 and 1:1. All samples were incubated at room temperature for 10 minutes and then 20 μL of each sample were applied to a carbon coated copper grid. After 5 min the grids were washed with sterile water, stained with 2% (w/v) uranyl acetate for 1 minute and washed again with water. Images were collected on a Jeol 1200 microscope (Boston, MA, USA) operating at 80 kV.

### Dynamic light scattering

The hydrodynamic radius (Rh) of samples at a rPrP to RNA ratio of 20:1, aggregated in buffer A for 10 minutes at room temperature was evaluated using DynaPro Nanostar (Wyatt, USA), equipped with a 662 nm laser in quartz cuvette at 25 °C. The DLS data for each sample were collected one to three times, with ten cumulates in each measurement, the mean of which lead to respective Rh values. Population distributions were analyzed by correlation function graphs.

### Circular dichroism

rPrP (5 μM) was mixed with domain V rRNA, DHFR mRNA and GFP+ mRNA to 1:0.05 ratio (rPrP:RNA) in buffer A and incubated from 5 to 10 min at room temperature before each measurement. CD spectra were collected with a Chirascan CD Spectrometer (Applied Photophysics, Surrey, UK) from 250 to 200 nm at 70 nm/min rate using a 1 mm-path length quartz cuvette. The spectra for protein and RNAs alone at their respective final concentrations were recorded separately.

## Data Availability

The data used in this manuscript are added in the result section, especially in the figures and tables.
